# A Case Report of SYNE1 Deficiency-Mimicking Mitochondrial Disease and the Value of Pangenomic Investigations

**DOI:** 10.3390/genes14122154

**Published:** 2023-11-29

**Authors:** Mounir Serag, Morgane Plutino, Perrine Charles, Jean-Philippe Azulay, Annabelle Chaussenot, Véronique Paquis-Flucklinger, Samira Ait-El-Mkadem Saadi, Cécile Rouzier

**Affiliations:** 1 Service de Génétique Médicale, Hôpital l’Archet 2, CHU de Nice, 151 Route Saint-Antoine de Ginestière, 06202 Nice, France; serag.m@chu-nice.fr (M.S.); plutino.m@chu-nice.fr (M.P.); chaussenot.a@chu-nice.fr (A.C.); veronique.paquis@unice.fr (V.P.-F.); saadi.s@chu-nice.fr (S.A.-E.-M.S.); 2 CNRS UMR7284/ INSERM U1081, Faculté de Médecine, Université Côte d’Azur, 06107 Nice, France; 3 Service de Génétique, La Pitié-Salpêtrière, AP-HP, 75610 Paris, France; perrine.charles@aphp.fr; 4 Service de Neurologie, 13005 Marseille, France; jean-philippe.azulay@ap-hm.fr

**Keywords:** WES, array-CGH, *SYNE1*, mitochondrial disorder

## Abstract

Mitochondrial disorders are characterized by a huge clinical, biochemical, and genetic heterogeneity, which poses significant diagnostic challenges. Several studies report that more than 50% of patients with suspected mitochondrial disease could have a non-mitochondrial disorder. Thus, only the identification of the causative pathogenic variant can confirm the diagnosis. Herein, we describe the diagnostic journey of a family suspected of having a mitochondrial disorder who were referred to our Genetics Department. The proband presented with the association of cerebellar ataxia, COX-negative fibers on muscle histology, and mtDNA deletions. Whole exome sequencing (WES), supplemented by a high-resolution array, comparative genomic hybridization (array-CGH), allowed us to identify two pathogenic variants in the non-mitochondrial *SYNE1* gene. The proband and her affected sister were found to be compound heterozygous for a known nonsense variant (c.13258C>T, p.(Arg4420Ter)), and a large intragenic deletion that was predicted to result in a loss of function. To our knowledge, this is the first report of a large intragenic deletion of *SYNE1* in patients with cerebellar ataxia (ARCA1). This report highlights the interest in a pangenomic approach to identify the genetic basis in heterogeneous neuromuscular patients with the possible cause of mitochondrial disease. Moreover, even rare copy number variations should be considered in patients with a phenotype suggestive of SYNE1 deficiency.

## 1. Introduction

Mitochondrial diseases are caused by defects affecting the mitochondrial oxidative phosphorylation pathway and genetic variants either in the mitochondrial genome (mtDNA) or nuclear DNA. They are considered a common cause of inherited metabolic disease, affecting approximately 1 in 5000 people [1]. The clinical spectrum is extremely heterogeneous, from severe syndromic neurological symptoms to pure isolated ones [1]. Cerebellar ataxia, either isolated or in conjunction with other symptoms, can be an outcome of mitochondrial disease, and features of mitochondrial dysfunction were found in 23% of patients with progressive ataxia who underwent muscle biopsy in the large study of Bargiela et al. [2]. The most common additional features were deafness, impaired glycemic control/diabetes, myoclonus, neuropathy, and spastic paraparesis. Mitochondrial disorders are also characterized by a high degree of genetic heterogeneity, with over 400 different genes involved. In adults, most mutated genes are involved in mtDNA maintenance, and patients might exhibit mtDNA deletions.

Furthermore, many genetic disorders can mimic mitochondrial disorders, and several studies have reported that more than half of patients suspected of mitochondrial disorder harbor pathogenic variants in non-mitochondrial genes [3,4]. These individuals may live for many years without receiving a diagnosis, impacting both their clinical assessment and treatment strategies. In our Reference Center for Mitochondrial Disorders CALISSON (www.mito-calisson.fr, accessed on 27 November 2023), since the implementation of whole exome sequencing (WES), we also identified pathogenic variants in non-mitochondrial genes in many patients initially suspected of having a mitochondrial disorder, including one family with pathogenic variants in the *SYNE1* gene. SYNE1 deficiency is one of the most common autosomal recessive ataxias. Spectrin repeat-containing nuclear envelope protein 1 (SYNE1) is one of the largest genes in the human genome, with 146 exons encoding Nesprin-1, which is a structural protein that anchors the nuclear membrane to the actin cytoskeleton [5]. So far, the phenotypic spectrum related to pathogenic variants in *SYNE1* ranges from ARCA1/SCAR8 with late-onset to the most severe condition, which is the arthrogryposis multiplex congenita [6,7,8,9,10,11,12]. ARCA1 is a slowly progressive disorder that commonly presents as either pure cerebellar ataxia (cerebellar ataxia, dysarthria, dysmetria, abnormalities in ocular saccades, and smooth pursuit) or is associated with an upper neuron dysfunction (spasticity, hyperreflexia, Babinski sign) and/or lower motor neuron dysfunction (amyotrophy, reduced reflexes, fasciculations), in some cases with cognitive impairment [11,13,14,15,16]. Most pathogenic variants are nonsense or frameshift and are localized throughout the gene, excluding the KASH (Klarsicht/ANC-1/Syne-1 homology) domain. Most pathogenic variants associated with motor neuron involvement are located toward the 3’ end of the gene [16].

Herein, we described the diagnostic journey of a family with a phenotype evocative at first of a mitochondrial disease in whom we identified biallelic pathogenic *SYNE1* variants, including the first large deletion reported in the *SYNE1* gene in an autosomal recessive cerebellar ataxia (ARCA1) phenotype. This case report highlights the clinical and diagnostic challenges associated with heterogeneous neuromuscular diseases. 

## 2. Materials and Methods 

### 2.1. Patients

**Figure 1:**
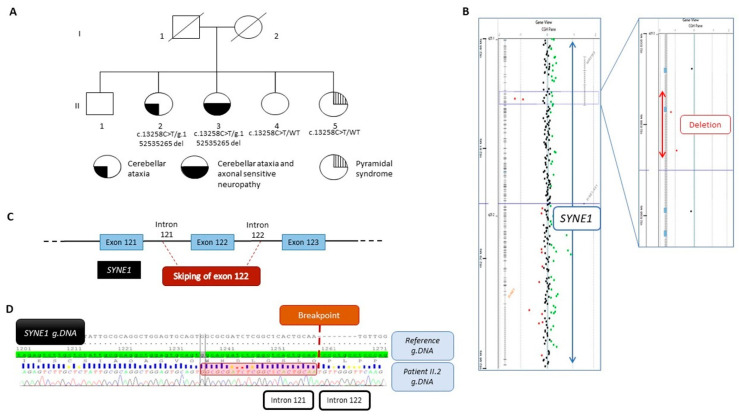
(**A**) Pedigree of the family and segregation analysis of the *SYNE1* variant NM_182961.4:c.13258C>T and the deletion NC_000006.11:g.152538134_152535265del. The filled bottom left quarter represents cerebellar ataxia symptom, the filled bottom right quarter represents axonal sensitive neuropathy, and the striped top quarter represents pyramidal syndrome. (**B**) View from Cytogenomic software (v.4.0.3.12) of *SYNE1* with a focus on the two deleted oligonucleotides (red dots). (**C**) Schematic view of the deletion encompassing the exon 122 and part of intron 121 and intron 122. (**D**) Representative Sanger sequencing electrophoregrams illustrating the breakpoint between intron 121 and intron 122. The 23 bp homology sequence is shown in the red square.

The proband, II-2, was born from non-consanguineous parents (Figure 1A). She developed a slowly progressive pure cerebellar ataxia with onset at thirty years of age. At 50 years old, muscle biopsy histology analysis showed atrophic type II fibers with few cytochrome c oxidase (COX) negative fibers. MtDNA analysis using both a long-range PCR and Southern blot showed multiple mtDNA deletions. Cerebral MRI showed cerebellar atrophy. At 60 years old, she had bradycardia, requiring a pacemaker. 

Her sister, II-3, had a similar phenotype. She presented with cerebellar ataxia at the age of 23 years. At 50 years, she needed a walker, and at 65 years, a wheelchair. On examination, there was a static and kinetic cerebellar syndrome of the four limbs with a SARA ataxia score of 30/40 and a very debilitating cerebellar action tremor. Eye pursuit was saccadic with nystagmus. Achilles reflexes were limited, and there was ankle hypopallesthesia. 

The youngest sister, II-5, had had pyramidal syndrome since the age of 47, without ataxia. At 50 years old, muscle biopsy histology showed only denervation abnormalities. 

We performed genetics analysis on siblings II-2, II-3, II-4, and II-5. All participants provided informed consent before enrolment. A local ethical committee approved the experimental protocol (Protocol 2018-A01903-52).

### 2.2. Whole Exome Sequencing (WES)

Genomic DNA (gDNA) was extracted from whole blood using a Chemagic 360 (Perkin Elmer™). Whole exome targets were captured and enriched using the SureSelect Agilent in-solution enrichment methodology with their biotinylated oligonucleotides probe library (Agilent, Santa Clara, CA, USA), and then sequenced on the Illumina HiSeq2000 (Illumina, San Diego, CA, USA) as paired-end, 75-base reads. Bioinformatics analysis were performed using the Illumina pipeline. Sequence reads were mapped to the human genome build (hg19/GRCh37). Variant calling was performed to call single-nucleotide variants (SNVs) and short insertions/deletions. 

Detected variants were annotated, filtered, and prioritized using the Cartagenia Bench NGS Lab (Agilent, Santa Clara, CA, USA). To prioritize disease-causative variants and single-nucleotide variants (SNVs) in coding regions and intron–exon junctions and short indels were excluded when the minor allele frequency (MAF) was >1% for in-house exomes, gnomAD (the Genome Aggregation Database), and other databases (dbSNP, 1000 Genomes, ExAC). Genes known to be associated with autosomal recessive cerebellar ataxia were selected, and variant segregation analysis using Sanger sequencing was performed in the family to identify the candidate gene.

### 2.3. Sanger Sequencing

Amplicons were amplified with a 5′-GAGATAGAGTCTTGCTATTG-3′ forward primer and a 5′-TGCTGTTCCAAAAGGTGCTG-3′ reverse primer. Thermal cycling conditions for PCR consisted of denaturation at 93 °C for 4 min, followed by 20 cycles of 93 °C for 10 s, 62 °C for 30 s, and 68 °C for 13 min; then 10 cycles of 93 °C for 10 s, 62 °C for 30 s, and 68 °C 13 min + 20 sec/cycle; and finally one cycle at 72 °C for 10 min. PCR products were purified with the Illustra ExoProStar enzyme (GE Healthcare, Little Chalfont, UK), processed with a BigDye™ Terminator Cycle Sequencing Kit (Thermo Fisher, Foster City, CA, USA) and analyzed on an ABI3130XL automated sequencer (Thermo Fisher). 

### 2.4. High-Resolution Array-CGH

High-resolution array comparative genomic hybridization (array-CGH) analysis was performed on the patient’s genomic DNA. DNA digestion, labeling, and hybridization were performed according to the manufacturer’s protocols (Agilent). DNA specimens were analyzed with the Human Genome CGH Microarray Kit and Microarrays Sureprint G3 Human 1X1M (Agilent Technologies™, Santa Clara, CA, USA), with an average space of 3.1 kb. Microarrays were scanned using an Agilent scanner, and image files were processed using Agilent’s Feature Extraction software (v.4.0.3.12) Data were visualized using Agilent’s Cytogenomics^®^.

### 2.5. Quantitative Real-Time PCR Assay

A confirmation of array-CGH results was performed using quantitative real-time PCR (qPCR) and the SYBR Green gene expression assay on a Light Cycler 400 platform (Roche^®^, Bale, Switzerland). The provided qPCR primer sequences were designed using UCSC database sequences of the region between two consecutive deleted probes (chr6: 152,537,544-152,536,208). The forward primer was 5′-TGATTGAGTCTCACCAGCTGT-3′, and the reverse primer was 5′-CCTCCTTGGGTTCTTGCTAGA-3. Additionally, the labeling for qPCR was conducted using Light Cycler 480 SYBR^®^ Green Master without ROX. Thermal cycling conditions for qPCR consisted of initial denaturation at 95 °C for 5 min, followed by 40 cycles at 95 °C for 15 s, 60 °C for 18 s, and 72 °C for 35 s. 

## 3. Results 

### 3.1. Sequencing Analysis

Because of a phenotype compatible with mitochondriopathy-associated neurologic features, mtDNA deletions and COX-negative fibers in the muscle, pathogenic variants in nuclear genes associated with mtDNA instability, including *POLG*, were first ruled out. Exhaustive mtDNA sequencing from the muscle also failed to identify a pathogenic variant. 

Then, a few years later, WES was performed, and data analysis in patient II-2 did not show deleterious variants in nuclear genes involved in mitochondrial disorders but allowed the identification of a heterozygous nonsense variant in exon 78 of *SYNE1* (NM_182961.4:c.13258C>T, p.(Arg4420Ter)). The affected sister, II-3, and both other sisters, II-4 and II-5, were also heterozygous for this variant. Using the strategy filtering described in the Materials and Methods section and variant segregation analysis in all family members, *SYNE1* was the only variant identified that made sense. The affected sister, II-3, and both other sisters, II-4 and II-5, were also heterozygous for this variant. This variant was reported as pathogenic in ClinVar (RCV002549102) and was previously reported in a family with ARCA1 [17]. It was classified as pathogenic according to the ACMG classification [18]. No further rare variants were found in this gene. 

### 3.2. High-Resolution Array-CGH and Familial Segregation Analysis

Because the phenotype strongly suggested SYNE1 involvement and the recessive mode of inheritance was suspected, we assumed that a variant might be missed by WES. To identify a second hit, we performed high-resolution array-CGH in the proband, II-2, and identified a loss of signal intensity for two consecutive probes located on chromosome 6q25.2 between positions 152,537,544 and 152,536,208 (Hg19) (Figure 1B). Quantitative PCR analysis confirmed this deletion to be heterozygous. Additional experiments using PCR and Sanger sequencing led us to identify a deletion of 2869 bp, involving exon 122 and a part of introns 121 and 122 (Figure 1C), as described below, according to the following ISCN 2020 and HGVS recommendations: seq[GRCh37] del(6)(q25.2q25.2) NC_000006.11:g.152538134_152535265del (Figure 1D). In silico analysis aided by the use of AnnotSV [14] predicted the deletion to be likely pathogenic. Expasy software (v.3.0) predicted that the deletion resulted in a truncated protein at position 7414 out of 8797 amino acids, i.e., 1383 amino acids shorter. Familial segregation analysis also showed the deletion in patient II-3, but no deletion was evident in both younger siblings, II-4 and II-5, confirming the compound heterozygous status of patients II-2 and II-3. At both boundaries of this deletion, a 23 bp homology sequence was identified, suggesting that the deletion may have originated from a non-homologous end-joining (NHEJ) repair mechanism (Figure 1D).

## 4. Discussion 

The identification of two pathogenic variants in *SYNE1* was consistent with the clinical features presented by both sisters II-2 and II-3, with ataxia beginning in the third decade and cerebellar atrophy. These results allowed us to correct the misdiagnosis of mitochondrial disease and confirmed the clinical diagnosis of ARCA1 [13]. The *SYNE1* gene encodes spectrum repeat-containing protein expressed in the skeletal and smooth muscle and peripheral blood lymphocytes that localize to the nuclear membrane. It is a multi-isomeric modular protein that forms a linking network between organelles and the actin cytoskeleton to maintain the subcellular spatial organization. Up to this point, there have been no reported connections with the mitochondrial metabolism. Additionally, considering the patient’s age during the biopsy, the presence of a limited number of COX-negative fibers and multiple mtDNA deletions were highly likely to be associated with the aging process. 

**Ataxia in mitochondrial disorders**

Mitochondrial disorders are known for their large clinical heterogeneity and the high number of phenocopies that make them difficult to recognize. Thus, only the identification of the causative mutation can confirm the diagnosis. Cerebellar ataxia is one of the main clinical features presented by patients referred to our Reference Center for Mitochondrial Disorders. Patients can have pathogenic variants in mtDNA genes, in disorders such as the NARP syndrome (neuropathy, ataxia, and retinitis pigmentosa) or Kearns–Sayre syndrome due to a large-scale heteroplasmic mtDNA deletion. It is also a classical sign of disorders with mtDNA instability; these patients typically exhibit multiple deletions of mtDNA in the muscle. More than twenty genes involved in mtDNA maintenance are implicated in mitochondrial disease [19]. The vast majority of them involve proteins with a role in the mtDNA replisome (*POLG* and *POLG2*, *TWNK*, *DNA2*, *MGME1*), including the supply of dNTP for mtDNA synthesis (*TP*, *TK2*, *DGUOK*, *RRM2B*, *SUCLA2*, *SUCLG1*) or regulating mitochondrial dynamics (*OPA1*, *MFN2*, *CHCHD10*) [19,20,21]. The genes most frequently involved in ataxias are POLG, which is responsible for the SANDO syndrome (sensory neuropathic ataxia with dysarthria and ophthalmoplegia); *TWINKLE*, which is responsible for the IOSCA syndrome (infantile-onset spinocerebellar ataxia), and *SPG7*, which is responsible for CPEO/ptosis and spastic ataxia or progressive ataxic disorder [2,19,22]. For this last gene, the mechanism leading to multiple deletions is still unclear. 

**Mitochondrial hallmarks of aging**

With aging, mitochondrial functions undergo alterations, which are manifested in particular by a deficiency of the respiratory chain and damage to mtDNA due to the accumulation of reactive oxygen species (ROS). Both mtDNA deletions and COX-negative fibers are indicative of age-related mitochondrial dysfunctions [23,24]. 

As it is often difficult to interpret whether mtDNA deletions are linked to age or pathogenic variants in genes involved in stability, tools have been developed to better understand the molecular mechanisms underlying the formation of mtDNA deletions. For example, Lujan et al. created the LostArc method for quantifying deletions in circular mtDNA molecules and delineating their position, length, and sequence context in human tissue samples [25]. Bris et al. identified specific criteria distinguishing mtDNA rearrangements related to aging from those related to mtDNA maintenance using an in-depth analysis of mtDNA sequences from patients with mitochondrial disease and controls; these criteria were obtained using NGS before processing through eKLIPse [26]. The systematic use of such tools allows for better diagnostic guidance for these patients. Finally, we must remain vigilant regarding techniques based on a long-range polymerase chain reaction (PCR) [27]. To date, long-range PCR and Southern blot are classically used for mtDNA screening, but PCR can lead to amplification bias and can overestimate the presence of mtDNA deletions by preferentially amplifying the shortest species with respect to the full-length wild-type molecule, and Southern blot is hampered by limitations in speed and resolution. To avoid bias, PCR-free technologies have been developed, such as the hybridization-based capture of mtDNA and short-read sequencing NGS or technologies based on long reads. Long-read sequencing allows the sequencing of several kilobases in length and has also been shown to be effective in deleting structural alterations affecting the mitochondrial genome [28].

In the same way, the presence of COX-negative fibers must always be interpreted according to the age of the patient, and a quantitative analysis to be considered significant. The characteristic histological abnormalities of mitochondrial disorders are the ‘ragged-red’ fibers, which indicate an accumulation of the mitochondria within skeletal muscle, and the COX-negative fibers that show absent or reduced cytochrome c oxidase activity [29]. The percentage of such fibers varies greatly among patients with primary mitochondrial disorders. Collins et al. reported RRFs and COX-negative fibers ranging from <1–7% to 1–23%, respectively, in their patients with progressive external ophtalmoplegia [30]. However, neither the presence of RRF nor COX deficiency is specific to primary mitochondrial disorders. They are also observed in the general population and increase with age. RRFs are typically never observed before the fourth decade, and COX-negative fibers similarly occur at extremely low levels but increase exponentially thereafter with age [31]. They have also been reported in other neuromuscular disorders, such as muscular dystrophies, inclusion-body myositis, or an adult-onset acid maltase deficiency [32,33]. In the classification proposed by Bernier et al., which is considered to be major criteria, the percentages have to be greater than 2% for RRF, greater than 2% before the age of 50, and greater than 5% after the age of 50 for COX-negative fibers [34].

**Toward a pangenomic approach in mitochondrial disorders**

Due to the clinical and genetic heterogeneity linked to mitochondrial disorders, several studies have highlighted the advantages of WES instead of targeted panel sequencing in patients with heterogeneous neuromuscular diseases with a potential mitochondrial etiology [3,35,36]. Recently, a study based on a whole genome sequencing (WGS) approach reported that 63% of patients suspected of having a mitochondrial disorder were ultimately diagnosed with a non-mitochondrial disorder [4]. In the French cohort, reported by the MitoDiag network (https://www.mitodiag.fr/home, accessed on 24 November 2023), among the 68 patients with a possible mitochondrial disorder (mitochondrial disease criteria (MDC) score ≤ 4) and confirmed diagnosis [37], more than half carried pathogenic variants in non-mitochondrial genes, i.e., none were included in Mitocarta V3 (Human MitoCarta3.0 (broadinstitute.org)) (64% of children (32/50) and 67% of adults (12/18)). Due to the clinical overlap of neurogenetic syndromes, several articles have also highlighted the interest of the WES approach in patients with ataxia after eliminating the more common trinucleotide repeat neurometabolic disorders, with diagnostic yields of around 50–60% [38,39]. This strategy reduces diagnostic delays and saves time for the initiation of potential treatment.

Despite technical advances and database implementation, diagnostic rates are reaching their limit using current WES-based methods [39]; in this report, we also illustrate the potential of a pangenomic approach and identify the first large deletion of the *SYNE1* gene in a family with the ARCA1 phenotype. To our knowledge, only one larger deletion encompassing *SYNE1* with other vicinity genes (MYCT1, VIP, FBOX5, MTRF1L, and RGS17) has been reported as pathogenic in ClinVar (accession number: VCV000538429.1) in a patient suffering from Emery–Dreifuss muscular dystrophy type 4. We do not have information on any associated pathogenic variant that could explain this phenotype to be classically caused by missense pathogenic variants. It is likely that deletions of this gene were underestimated due to technical difficulties associated with targeted panel sequencing or WES. Nevertheless, large deletions appear to be infrequent occurrences. In a comprehensive cohort study conducted by Synofzik et al., which encompassed 434 patients with ataxia, including 50 individuals who underwent copy number variation (CNV) analysis targeting the 146 exons of *SYNE1*, no large deletions were detected [13]. Over the last few years, within the MitoDiag network, NGS technology has been sequentially and successively implemented with WES, gradually replacing resequencing gene panels in the diagnostic strategy of mitochondrial disorders. More recently, the implementation of WGS within both the national genomic sequencing platforms SEQOIA and AURAGEN has facilitated access to WGS and could potentially improve the detection of such deletions. Nevertheless, WGS remains a costly option and may not be accessible in all laboratories, making array-CGH another potentially valuable alternative.

This report crystallizes the need for clinicians to consider a broad range of potential diagnoses when a mitochondrial disorder is suspected and the need to undertake comprehensive and cutting-edge genetic analyses in order to establish an accurate diagnosis. Furthermore, even if rare, copy number variations should be considered in patients with a phenotype suggestive of a SYNE1 deficiency.

## Data Availability

Data are contained within the article.
